# Protective Effect and Possible Mechanisms of Tripterygium Glycosides in Patients with Ankylosing Spondylitis: A Systematic Review and Meta-Analysis

**DOI:** 10.1155/2022/9374895

**Published:** 2022-03-03

**Authors:** Zhou Lin, Mangmang Chen, Xuewu Chen, Jiaru Chen, Wei Zhang

**Affiliations:** Department of Orthopaedic Surgery, Wenzhou Central Hospital, Wenzhou, Zhejiang 325000, China

## Abstract

**Objective:**

The safety and efficacy of Tripterygium glycosides (TG) were assessed for ankylosing spondylitis (AS) in accordance with the existing literatures.

**Materials and Methods:**

Electronic literature was searched from Chinese VIP databases, Cochrane Library, Chinese Biomedical Literature Database, Wanfang Web of Science, EMBASE, Chinese National Knowledge Infrastructure, and the PubMed for the studies with the publication from the beginning to December 2021. Randomized controlled trials (RCTs) were included only. The major variables of result comprised erythrocyte sedimentation rate (ESR), C-reactive protein (CRP), Spinal Pain Visual Analog Score (SP-VAS), Bath Ankylosing Spondylitis Functional Index (BASFI), and Bath Ankylosing Spondylitis Disease Activity Index (BASDAI). Moreover, the secondary variables of result covered the overall clinical effective rate following the adverse drug reaction (ADR). We carried out the meta-analysis with the use of STATA 12.0 and RevMan 5.3. We used GRADE pro3.6.1 software to assess the quality of evidence.

**Results:**

In general, we covered 15 randomized controlled trials with the focus of 1186 patients. As proven by our meta-analysis, TG as adjuvant therapy or monotherapy decreased the BASDAI, BASFI, SP-VAS, serum CRP, and ESR than control in patients suffering from AS. Additionally, TG treatment visibly improved the overall effective rate in AS. Nevertheless, TG was not found to significantly increase the rate of ADR in contrast to the control.

**Conclusion:**

As indicated by our result, TG may be an option to treat AS. In this paper, we recommended strict trials with high quality and large samples sizes for confirming the finding here.

## 1. Introduction

Ankylosing spondylitis (AS) refers to a chronic disease that can cause inflammation. AS can result in damage of structures and inflammation, mostly occurring within the spine or the sacroiliac joint, which normally leads to patients' morning stiffness and chronic back pain and thus causes spinal immobility and ankylosis [1, 2]. In some severe patients, it ultimately could lead to the rigid and completely fused spine. “Bamboo spine” is formed based on ossification within the fibrous ring's external fibers of intervertebral discs, forming adjoining vertebra's marginal syndesmophyte [3]. Besides, it frequently gives rise to extra-articular aggravation such as inflammatory bowel disease, psoriasis, and uveitis [4]. Normally, young men with 20 to 30 years old are more prone to AS [5]. The estimation of over 90% genetic role was achieved, the most significant correlation with human leukocyte antigen (HLA)-B27 [6]. Nevertheless, HLA-B27's specific pathogenic effect is still elusive though many hypotheses are proposed.

Currently, AS treatment strategy has been optimized remarkably in past decades by employing tumor necrosis factor- (TNF-) specific agents, disease-modifying antirheumatic drugs (DMARDs), nonsteroidal anti-inflammatory drugs (NSAIDs), and some biologics [7]. Nevertheless, under NSAIDs therapy in the long run, the possible risk covering risk increases in the cardiovascular system, the gastrointestinal tract, and kidney [8]. DMARDs, including sulfasalazine (SSZ) and leflunomide (LEF), have been rarely recommended for treating axial spondyloarthritis, since they have no effect on axial spondyloarthritis, while playing limited roles in treating peripheral manifestations when coexisting with the axial disease [9]. Using DMARDs in a long term would have negative effects, for instance, anaphylaxis, gastrointestinal reaction, liver injury, and leukopenia, thus, limiting their use in clinical practice. TNF inhibitor treatment discontinuation within patients usually leads to 84.6% of partial remission and a relapse within 79.2%, termed responders, in accordance with ASAS40 criterion [10]. Moreover, the huge cost is also a disadvantage of biologics, which should be taken into consideration. As a result, novel therapeutic options or agents are needed for AS treatment.

In recent years, the rising application of alternative and complementary medicine, covering Chinese herbal medicine for AS treatment, arouses wide concern [11, 12]. *Tripterygium wilfordii* Hook, a woody vine pertaining to the Tripterygium genus, refers to a traditional Chinese medicine having anti-inflammation, antirheumatism, and immunomodulation impacts [13]. Tripterygium glycosides (TG), the main ingredients of *Tripterygium wilfordii* Hook, were employed in China to treat inflammatory diseases in the long run (e.g., AS, chronic nephritis, a wide variety of skin disorders, and rheumatoid arthritis) [14]. TG can achieve anti-inflammatory effect, collateral dredging effect, swelling subsidence effect, detoxification, dampness elimination effect, wind dispelling effect, and inhibition effect on humoral and cell immunity [15, 16]. TG has been demonstrated with the improvement effect of the clinical characteristics and the regulation effect of AS patients' serum biomarker [17]. Over the past few years, increasing randomized controlled trials with high quality proved TG safety and effectiveness in terms of AS treatment. Nevertheless, there has been rare meta-analysis or systematic review on TG safety and efficacy for AS treatment. For this reason, a systematic review and meta-analysis covering randomized controlled trials with high quality were conducted for assessing TG safety and effect for treating AS.

## 2. Methods

We conducted a systematic review and meta-analysis in accordance with the AMSTAR (assessing the methodological quality of systematic reviews) [18] and PRISMA (Preferred Reporting Items for Systematic Reviews and Meta-Analyses) guidelines. There are no protocols preregistered for this review. This paper was registered in Research Registry (https://www.researchregistry.com/), with reviewregistry1276 as the registration number. Major personal data was not acquired, so we did not need ethical approval. Some methods in this section referred to our previous study [19].

### 2.1. Database and Searching Strategy

The electronic search was conducted in 8 repositories, i.e., Chinese VIP Database, Cochrane Library, Chinese National Knowledge Infrastructure, EMBASE, Wanfang Database, Web of Science, Chinese Biomedical Literature Database, and PubMed, from their beginning to December 2021. Besides, additional related literatures were manually searched in existing systematic reviews' references. Besides, the searching of existing literatures had no limitation of publication language. The strategy for searching within the English database covered: ([“Tripterygium wilfordii Hook F”] OR [“Tripterygium wilfordii”] OR [“Tripterygium glycosides”] OR [“Tripterygium”] OR [“thunder god vine”]) AND ([“Ankylosing spondylitis”] OR [“AS”]) AND ([“random control trials”] OR [“randomized controlled trial”]). For the Chinese databases, we employed free text terms, covering ([“lei gong teng (i.e., Tripterygium wilfordii Hook F in Chinese)”] OR [“lei gong teng duo gan (i.e., Tripterygium glycosides in Chinese)”]) AND “qiang zhi xing ji zhu yan (i.e., ankylosing spondylitis in Chinese)” AND “sui ji dui zhao shi yan (i.e., randomized controlled trial in Chinese)”. The detailed search strategy used for English databases and Chinese databases was provided as supplementary material (available here).

### 2.2. Eligibility Standards

The studies were covered in accordance with the PICOS criterion below:

#### 2.2.1. Types of Participants

In this paper, we included patients diagnosed with AS [20] regardless of gender, age, severity, and course of disease.

#### 2.2.2. Types of Interventions

The examined treatment intervention was TG as monotherapy or supplementary treatment with western conventional medication, irrespective of the therapy's administration period, administrated methods, administration route, duration, or dosage.

#### 2.2.3. Types of Controls

The control was given western conventional medication. We excluded studies involving controls of Chinese herbal medicine treatments.

#### 2.2.4. Types of Result Measures

The primary outcome measures covered (1) SP-VAS (Spinal Pain Visual Analog Score), (2) BASFI (Bath Ankylosing Spondylitis Functional Index), (3) BASDAI (Bath Ankylosing Spondylitis Disease Activity Index), (4) ESR (erythrocyte sedimentation rate), and (5) CRP (C-reactive protein). ADR (adverse drug reaction) and the clinical effective rate (ER) were the secondary outcomes.

#### 2.2.5. Types of Studies

We only involved randomized controlled trials investigating the safety and efficacy of Tripterygium glycosides (TG) for AS with no limit of publication states or languages. If this paper searched an article that contained 3 treatment arms, we merely extracted data for the control arm (s) as well as the arm (s) that entails TG. Quasi-randomized trials with researches allocating subjects were excluded according to the admission number order and the date of birth.

### 2.3. Exclusion Standards

(1) Nonrandomized controlled trial; (2) randomized controlled trials in which the patients were not reported to suffer from AS; (3) the combination of TG treatment and other drugs; (4) consistent animal experiments, reviews, and abstracts; (5) the duplication of publications and randomized controlled trials containing not sufficient data.

### 2.4. Literature Selection

In this paper, we used the flow diagram of PRISMA to choose the involved articles. The result of literature was introduced to the Endnote X7 software. 2 authors evaluated the potential eligible studies independently after they screened the title and abstract for the removal of duplication and not relevant research or the randomized controlled trials that were not involved in the inclusion standards. Afterward, we obtained the full text of the rest potential researches and reviewed these researches. A third independent investigator would resolve the disagreement between the first 2 authors.

### 2.5. Data Abstraction

2 reviewers searched the data independently. Next, the uniformity was examined by a third independent reviewer. We used a standard form containing the retrieved items, which covered the general information of studies: the author's (s) name(s), publication date, design of study, patient gender and age, number of sample, intervention approach covering TG alone, or supplement western conventional medication and course of treatment. For continuous outcomes, we extracted mean and standard deviation (SD) as well as participant number of each research. For dichotomous outcomes, the total number and the number of events of both groups were extracted. Under probable conditions, we recalculated the data according to other types of form, as an attempt to carry out pooled analysis. Discussions would be conducted to solve the disagreement of the mentioned 2 reviewers. If necessary, we established contact with the involved studies' authors to acquire the missing data or additional data.

### 2.6. Evaluation of the Quality of Included Studies

With the use of the Cochrane collaboration tool, 2 authors independently evaluated the method quality and the bias risk in the included randomized controlled trial studies [21]. The above Cochrane tool is capable of evaluating incomplete result data, selective result reporting, result evaluation blinding, blinding of subjects, allocation concealment, randomization, and other types of biases in terms of the respective item. It can also classify studies into high risk of bias, low risk of bias, or not clear level of bias.

### 2.7. Evidence Quality Assessment

We evaluated the quality of the evidence according to the GRADE [22, 23]. We classified the meta-analysis results into high evidence quality, moderate evidence quality, low evidence quality, or very low evidence quality. First, we classified the randomized controlled trial results into evidence with high quality. The quality of each result decreased due to indirectness, publication bias, inconsistency, imprecision, and risk of bias. We adopted GRADE pro3.6.1 for the investigation and synthesis of data.

### 2.8. Statistical Analysis

The data were collected and input into the STATA software (V.12.0; StataCorp, College Station, TX) for meta-analysis. The bias risk assessment was evaluated by RevMan software version 5.3 (Cochrane Collaboration, Oxford, UK). For continuous outcomes, mean, standard deviation (SD), and sample number of each group were extracted. For dichotomous outcomes, the total number and the number of events of both groups were extracted. Standard mean difference (SMD) with 95% confidence interval (CI) was calculated to analyze continuous variables (ESR, CRP, SP-VAS, BASFI, and BASDAI). Relative risk (RR) with 95% CI was calculated for dichotomous variables (overall effective rate and ADR). Statistical heterogeneity among trials was assessed using the chi-squared test and *I*^2^ statistic. The heterogeneity among studies was identified if the *I*^2^ was greater than or equal to 50% or *P* value was less than or equal to 0.05. A random-effects model was applied when heterogeneity was detected or the statistical heterogeneity was high (*P* < 0.05 or *I*^2^ > 50%), and then, further subgroup study and meta-regression analysis (the number of included studies was more than 8) were performed to detect the origin of heterogeneity. Otherwise, a fixed-effects model was used (*P* ≥ 0.05 or *I*^2^ ≤ 50%). Publication bias was assessed by using Begg's and Egger's linear regression test in the meta-analysis, and we considered *P* < 0.05 to be significant. Stabilities of synthetic results were evaluated with a visual assessment of sensitivity analyses. The method of omitting each study in sequence was used for sensitivity analysis.

## 3. Result

### 3.1. Study Description

**Figure 1:**
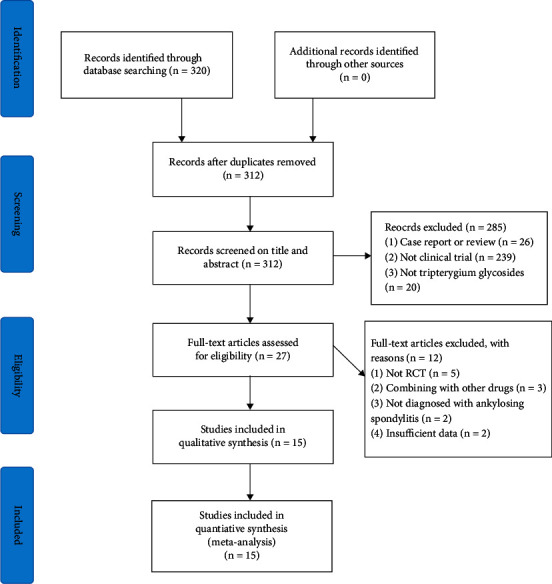
Flowchart of study selection.

We found 320 related studies in the databases searched. Afterward, 312 studies were left when duplicate removal was achieved. Subsequently, we removed 285 studies based on specific tittle and abstract screening. By screening 27 articles' full texts, we removed 12 studies due to the nonconformity with the study inclusion standards. Finally, 15 studies [24–38] were involved for investigation.  Figure 1 presents the search standards and the process of selection specifically.

### 3.2. General Characteristics of the Covered Studies

Table 1 lists the characteristic exhibited by all the included randomized controlled trials. The publication of the trails ranged from 2011 to 2021. In general, the randomized controlled trials covered 1186 patients, 569 within the control in comparison with 617 within the experiment group. The studies generally tested the impact arising from TG on AS, 8 studies [25, 31–34, 36–38] used sulfasalazine as the control, 3 studies [24, 29, 35] used leflunomide as the control, and 4 studies [26–28, 30] used etanercept as the control. The researches covered no less than 8 weeks of the intervention with the exception of 2 studies [32, 35]. They did not report the course of treatment in their studies.

### 3.3. Risk of Bias

**Figure 2:**
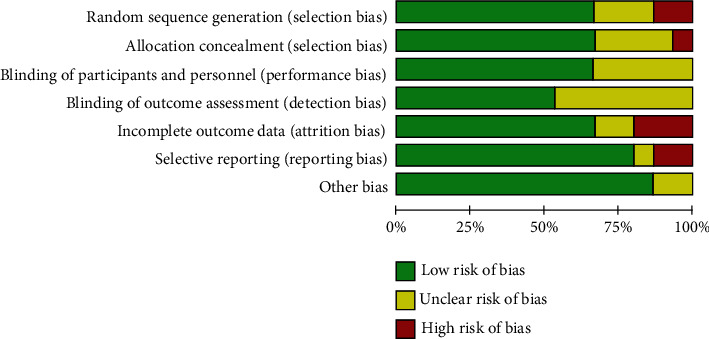
Risk of bias graph: review authors' judgements about each risk of bias item presented as percentages across all included studies.

**Figure 3:**
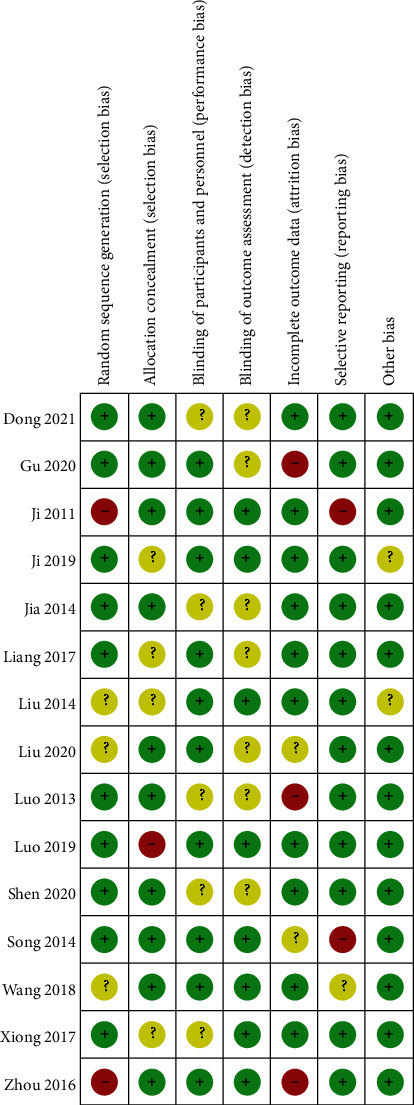
Risk of bias summary: review authors' judgements about each risk of bias item for each included study.

In this paper, we applied the Cochrane risk of bias tool for exploring the bias risk. According to the 15 included studies, the standard number had a range (4/7 to 6/7). Besides, 10 of the included randomized controlled trials [24, 25, 27–29, 31, 32, 34, 36, 38] presented the precise method used for generating random sequence. 10 studies [25–27, 30, 31, 33, 35–38] indicated the blinding, and the rest were still not clear. 10 studies [25, 27–29, 32–35, 37, 38] complied with the not complete result data standards because no drop-out patient or drop-out information was found. 2 studies [36, 37] contained bias risk within selective reporting. A baseline comparison was drawn, and the participants' consent was effectively documented. Furthermore, most studies included did not show other biases. More specific information regarding risk of bias assessment of the respective trial is presented in Figures 2 and 3.

### 3.4. Results of Meta-Analysis

#### 3.4.1. BASDAI

**Figure 4:**
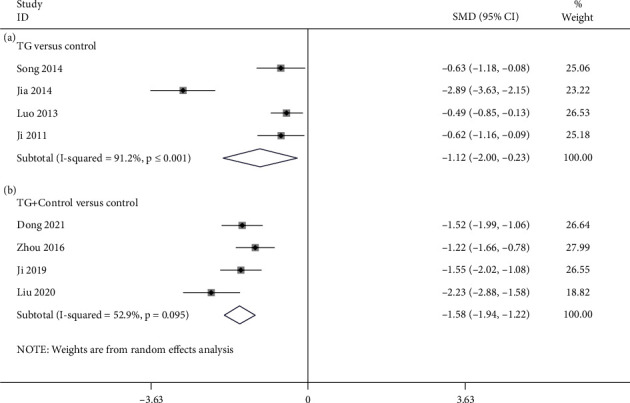
Forest plot of BASDAI: (a) TG versus control and (b) TG plus control versus control.

**Figure 5:**
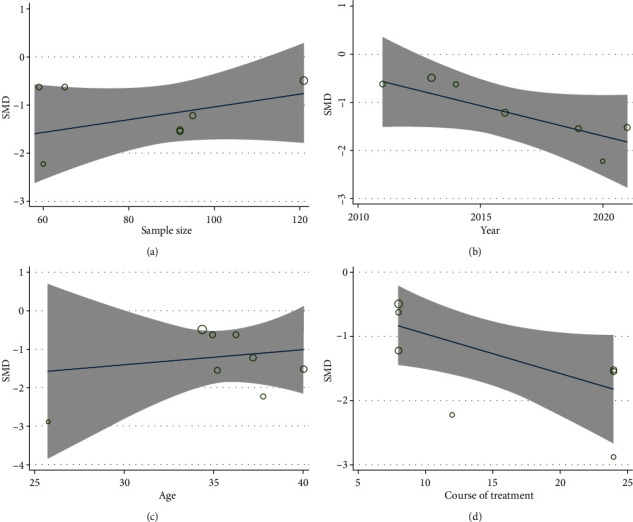
Meta-regression analysis of BASDAI. (a) Sample size. (b) Publication year. (c) Age. (d) Course of treatment.

4 studies [24, 29, 36, 37] compared TG alone with the control regarding BASDAI. According to Figure 4(a), as revealed by the pooled result, TG monotherapy was remarkable for reducing BASDAI in contrast to the control (SMD = −1.116; 95%CI = −2.001 to −0.232; *P* = 0.013; heterogeneity *χ*^2^ = 33.97, df = 3, *I*^2^ = 91.2%, *P* < 0.001). 4 studies [26, 30, 34, 38] reported TG plus control versus control in accordance with BASDAI. The pooled results illustrated that TG plus control had significance in terms of the reduction of BASDAI in contrast to the control (SMD = −1.578; 95%CI = −1.940 to −1.216; *P* < 0.001, heterogeneity *χ*^2^ = 6.37, df = 3, *I*^2^ = 52.9%, *P* = 0.095, Figure 4(b)). Meta-regression was used for the exploration of heterogeneity sources. To find the probable source of heterogeneity in studies, we carried out the meta-regression investigation in terms of sample size, year of publication, age, and treatment course (Figure 5). Overall, the course of treatment (*β* = −0.069; *P* = 0.04; Adj *R*^2^ = 42.53%) might be the major heterogeneity source. However, the sample size (*β* = 0.015; *P* = 0.302; Adj *R*^2^ = 3.66%), age (*β* = 0.087; *P* = 0.285; Adj *R*^2^ = 3.52%), and publication year (*β* = −0.107; *P* = 0.238; Adj *R*^2^ = 14.81%) were not the prominent heterogeneity sources for BASDAI.

#### 3.4.2. BASFI

**Figure 6:**
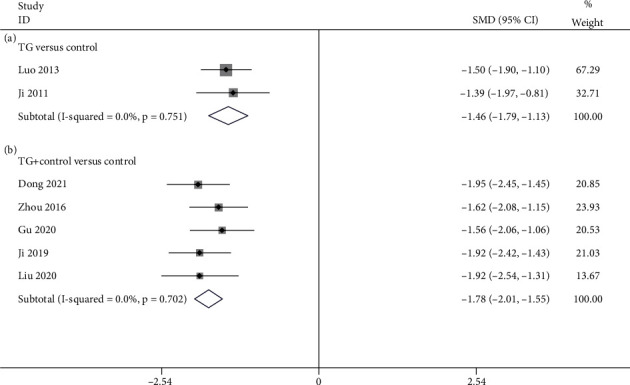
Forest plot of BASFI: (a) TG versus control and (b) TG plus control versus control.

2 studies [24, 37] drew the comparison between TG alone and the control for BASFI. The available data illustrated that TG monotherapy dramatically depressed the BASFI in contrast to the control (SMD = −1.463; 95%CI = −1.794 to −1.131; *P* < 0.001; heterogeneity *χ*^2^ = 0.10, df = 1, *I*^2^ = 0%, *P* = 0.751, Figure 6(a)). 5 studies [26, 30, 31, 34, 38] drew the comparison between TG plus control and the control about BASFI. As revealed by the pooled result, TG and western medicine combination noticeably reduced BASFI in contrast to western medicine alone (SMD = −1.781; 95%CI = −2.008 to −1.553; *P* < 0.001, heterogeneity *χ*^2^ = 2.18, df = 4, *I*^2^ = 0%, *P* = 0.702, Figure 6(b)).

#### 3.4.3. SP-VAS

**Figure 7:**
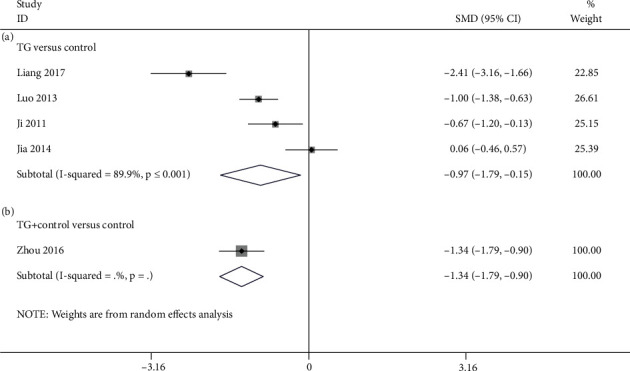
Forest plot of SP-VAS: (a) TG versus control and (b) TG plus control versus control.

4 studies [24, 25, 29, 37] reported TG alone versus control in accordance with SP-VAS. According to the pooled results, TG monotherapy had significance in terms of lessening SP-VAS in contrast to the control (SMD = −0.970; 95%CI = −1.793 to −0.147; *P* = 0.021, heterogeneity *χ*^2^ = 29.63, df = 3, *I*^2^ = 89.9%, *P* < 0.001, Figure 7(a)). Only one study [26] compared TG plus control with the control for SP-VAS. As revealed by the study, TG and western medicine combination significantly reduced SP-VAS in comparison with western medicine alone (SMD = −1.344; 95%CI = −1.791 to −0.898; *P* < 0.001, Figure 7(b)).

#### 3.4.4. CRP

**Figure 8:**
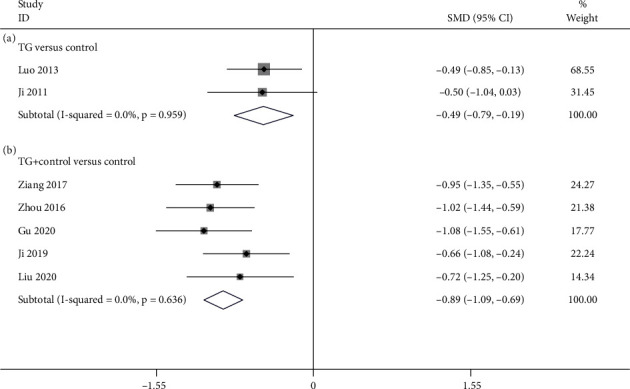
Forest plot of CRP: (a) TG versus control and (b) TG plus control versus control.

There were 2 studies [24, 37] that compared TG plus control with the control about CRP. As revealed by the pooled result, TG monotherapy markedly downregulated CRP level in contrast to the control (SMD = −0.492; 95%CI = −0.792 to −0.193; *P* = 0.001, heterogeneity *χ*^2^ < 0.001, df = 1, *I*^2^ = 0%, *P* = 0.959, Figure 8(a)). 5 studies [26, 30–32, 38] referred to TG plus control versus control on CRP. According to the pooled data, TG plus control distinctly decreased CRP in contrast to the control (SMD = −0.890; 95%CI = −1.088 to −0.692; *P* < 0.001, heterogeneity *χ*^2^ = 2.55, df = 4, *I*^2^ = 0%, *P* = 0.636, Figure 8(b)).

#### 3.4.5. ESR

**Figure 9:**
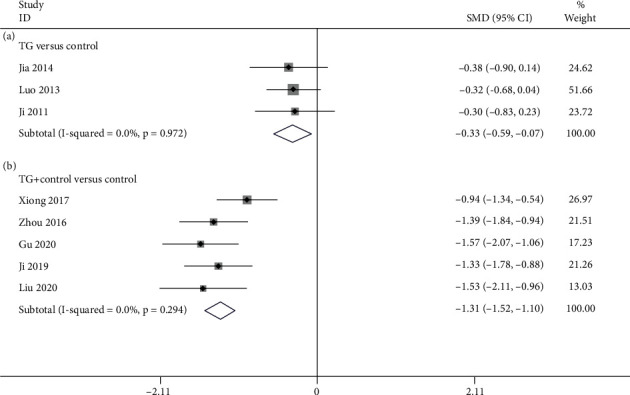
Forest plot of ESR: (a) TG versus control and (b) TG plus control versus control.

3 studies [24, 29, 37] compared TG alone with the control with regards to ESR. According to Figure 9(a), the pooled results exhibited that TG monotherapy was noticeable in lowering ESR in contrast to the control (SMD = −0.331; 95%CI = −0.588 to −0.073; *P* = 0.012; heterogeneity *χ*^2^ = 0.06, df = 2, *I*^2^ = 0%, *P* = 0.972). 5 studies [26, 30–32, 38] mentioned TG plus control versus control about ESR. As revealed by the pooled data, TG plus control notably reduced ESR in contrast to the control (SMD = −1.307; 95%CI = −1.515 to −1.098; *P* < 0.001, heterogeneity *χ*^2^ = 4.93, df = 4, *I*^2^ = 18.9%, *P* = 0.294, Figure 9(b)).

#### 3.4.6. ER

**Figure 10:**
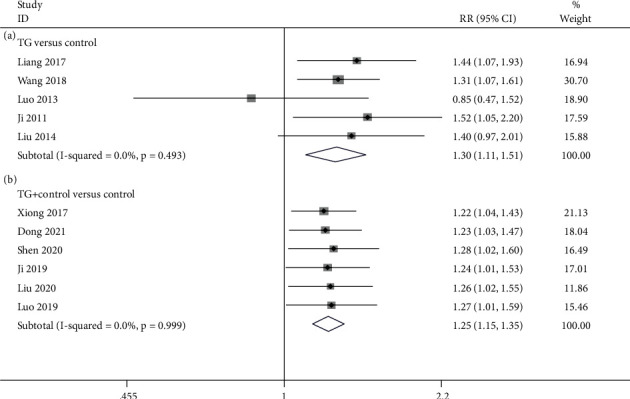
Forest plot of ER: (a) TG versus control and (b) TG plus control versus control.

There were 5 studies [24, 25, 33, 35, 37] comparing TG alone with the control about the effective rate. According to the pooled data, TG monotherapy evidently elevated effective rate in contrast to the control (RR = 1.295; 95%CI = 1.112 to 1.509; *P* = 0.001, heterogeneity *χ*^2^ = 3.40, df = 4, *I*^2^ = 0%, *P* = 0.493, Figure 10(a)). 6 studies [27, 28, 30, 32, 34, 38] referred to TG plus control versus control for the effective rate. The pooled data revealed that TG plus control obviously enhanced effective rate in comparison to the control (RR = 1.247; 95%CI = 1.150 to 1.353; *P* < 0.001, heterogeneity *χ*^2^ = 0.19, df = 5, *I*^2^ = 0%, *P* = 0.999, Figure 10(b)).

#### 3.4.7. ADR

**Figure 11:**
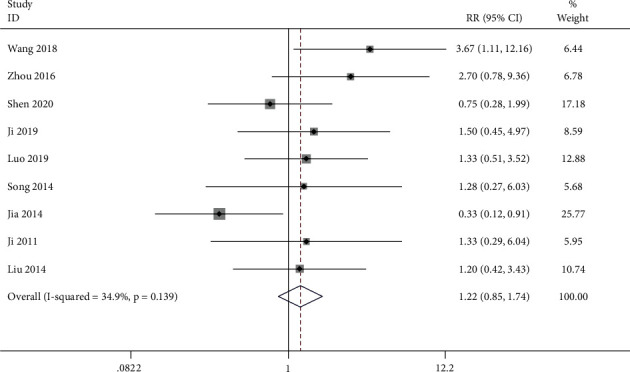
Forest plot of ADR.

In this paper, we found ADR from 9 studies [26–29, 33, 35–38]. The adverse event frequency reached 61/364 of the control and 45/315 of the trial group. According to the pooled data, the ADR rate reported insignificant difference in the 2 groups (RR = 1.216; 95%CI = 0.848 to 1.742; *P* = 0.288, heterogeneity *χ*^2^ = 12.28, df = 8, *I*^2^ = 34.9%, *P* = 0.139, Figure 11). As revealed by the results of this paper, amenorrhea, menstruation disorders, and liver function damage or gastrointestinal discomfort frequently constitute the most frequently occurring adverse events. Significant negative effects were mild, without any high negative effect, covering life threatening indicated by the covered randomized controlled trials.

### 3.5. Subgroup Analysis

We carried out subgroup investigation in accordance with the course of treatment, sample size, age, control medication, and TG dosage in Table 2 because of the significant heterogeneity of SP-VAS and BASDAI result in our paper. Nevertheless, according to the results of subgroup investigation, the mentioned factors were not the prominent heterogeneity source in terms of BASDAI and SP-VAS.

### 3.6. Publication Bias and Sensitivity Investigation

**Figure 12:**
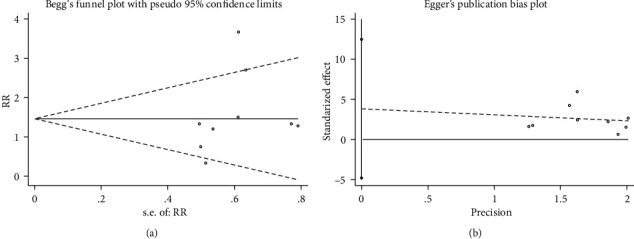
Begg's test and Egger's test of ADR.

In this paper, we performed the Begg's test and Egger's test (Figure 12) for examining ADR's possible publication bias within this meta-analysis. Consequently, according to the *P* values from Begg's test and Egger's test, ADR had no remarkable publication bias (*P* = 0.251 and *P* = 0.330, separately).

**Figure 13:**
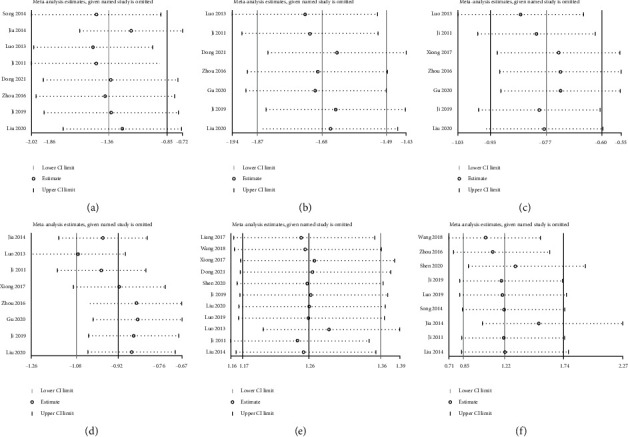
Sensitivity analysis for BASDAI (a), BASFI (b), CRP (c), ESR (d), ADR (e), and ER (f).

For establishing the impact arising from each involved studies relating to BASDAI, BASFI, CRP, ESR, ADR, and ER pooled data to demonstrate the findings here to be robust, the sensitivity was evaluated through the exclusion of one study at a time and computing the pooled data for other randomized controlled trials. According to the sensitivity investigation results, no noticeable effect was exerted on pooled data when the respective study was eliminated, respectively, thereby indicating the finding here to be relatively robust (Figure 13).

### 3.7. GRADE Assessment

We used the GRADE method for exploring the results' evidence quality, with moderate or low methodological issues and heterogeneity, as well as significantly low quality. According to Table 3, the evidence quality of 5 results was low, one result had very low evidence quality, and another result had moderate evidence quality. Thus, the overall evidence quality of this study was low.

## 4. Discussion

AS, the axial skeleton's chronic inflammatory disease, leads to the rigid and completely fused spine [39, 40]. Medications (NSAIDs and DMARDs) used to treat AS have been used in clinical practice for many years [8]. However, the diverse adverse reaction such as anaphylaxis, gastrointestinal reaction, liver injury, and leukopenia limit their long-term use. Tumor necrosis factor blockers refer to a promising drug to treat AS. Nevertheless, tumor necrosis factor blockers are likely to lead to some significant side effects, covering neurological issues and reactivating latent tuberculosis [41]. Therefore, safe and effective anti-AS drugs are urgently required. With the progress of clinical practice and research, increasing evidence reveals that Chinese herbal medicine has prominent effects in preventing and treating AS [14]. TG, extracted from Traditional Chinese Medicinal plant Tripterygium, has been used in China and other Asian countries for the long-term treatment of inflammatory diseases covering AS, chronic nephritis, different skin disorders, and rheumatoid arthritis [15]. According to modern pharmacological research, TG can achieve anti-inflammatory effect, collateral dredging effect, swelling subsidence effect, detoxification, dampness elimination effect, wind dispelling effect, and inhibition effect on humoral and cell immunity [15, 16]. Considerable randomized controlled trials have demonstrated that TG was advantageous in treating AS [27, 31, 34], whereas no high-quality systematic review and meta-analysis with large sample size on the safety and efficacy of TG to treat AS has been demonstrated. Thus, a systematic review and meta-analysis was carried out for the investigation of whether TG has safety and efficacy in patients suffering from AS to evidence clinical practice and scientific study.

### 4.1. Summary of Evidence

The current paper has been the initial systematic review and meta-analysis investigating whether TG monotherapy and TG supplied with western medicine have efficacy and safety to treat AS. On the whole, fifteen high-quality randomized controlled trials involving 1186 individuals with AS were included in the investigation. According to the major finding of existing systematic reviews and meta-analyses, using TG as adjuvant therapy or monotherapy for AS treatment evidently decreased BASDAI, BASFI, and SP-VAS, thereby demonstrating that TG can effectively relieve the pain that is caused by AS. Next, the serum CRP and ESR were lowered by TG. In addition, TG treatment visibly elevated the overall effective rate in AS. Yet, there was no significant difference in the incidence of adverse reactions between the 2 groups, thereby demonstrating that TG has good safety and tolerability for AS patients. Thus, supporting evidence was provided in our paper, showing that TG may be highly recommended for planned use in patients suffering from AS.

### 4.2. Comparison with Existing Studies

Some systematic reviews and meta-analyses demonstrated TG's efficacy and safety in AS treatment. As revealed by a meta-analysis [42] constituting 14 randomized controlled trials involving 996 patients, total effective rate, BASDAI, BASFI, and CRP showed no significant difference between the TG group and the western medicine group, inconsistent with the results of this paper. In their study, only 6 studies comparing TG with western medicine, and the rest 8 studies were compared with Chinese herbal compound. However, the studies with Chinese herbal compound control were excluded because numerous varieties of Chinese herbal compound of the control may result in high heterogeneity, and only the studies with western medicine control were involved. Thus, the sample size of this paper was significantly larger than theirs, which may be major cause of contradiction. Besides, part of the finding here was consistent with the data of Li et al. [3]. They performed a meta-analysis involving 11 trials that focused on 807 patients and exhibited that TG treatment obviously decreased the pain index, ESR in AS. Nevertheless, the CRP was not reduced in their study. The data here have no consistency with the findings of existing researches in several fields, probably correlated with (1) the included studies in the existing meta-analyses varied considerably in quality. Nonetheless, herein, randomized controlled trials having a risk of bias score ≥ 4 on the basis of the Cochrane RoB tool were included, implying that only high-quality randomized controlled trials were included in this paper. (2) The diversity and complexity of Chinese herbal compound in the control in the existing studies are also major causes of the difference. Nevertheless, herein, we focused particularly on the single western medicine in the control, which can reduce the risk of heterogeneity. (3) AS is a chronic condition with diverse stages. The AS distinct stages can affect the progress of the disease along with the treatment response. However, many existing studies and this paper have not reported the stages of AS, which may also lead to contradictions. (4) The prevalence of AS in young men is 10 times higher than that in women. Therefore, the gender ratio difference between existing studies and this paper also affects the research results. (5) The course of treatment may affect the outcome. The duration of the current and current studies was 8-24 weeks. It is a paradox that too short a course of treatment may lead to poor efficacy, while too long a course of treatment may lead to serious adverse reactions.

### 4.3. Strengths

The advantages of our meta-analysis studies include a clearly defined study issue, namely, reduced selection of randomized controlled trials with consistency, fidelity, and bias. A precise study methodology was designed prior to the meta-analysis and an in-depth search of the literature was conducted. 2 researchers evaluated the protocols for the input data components, as well as the quality control of all data. All of the studies covered in herein constituted randomized controlled trials with an extraordinary number being high quality, which contributes to overcoming the drawbacks of the recall or selection bias in terms of nonrandomized researches. In addition, the number of trials and overall sample size was large (15 trials, 1186 participants). Subgroup and meta-regression assessments were carried out for determining the source of heterogeneity. Therefore, no publication bias was reported in this meta-analysis, and sensitivity estimates suggest that the results of the present meta-analysis have relative robustness.

### 4.4. Limitations

However, this paper had some limitations. First, no protocol has been preregistered for our paper, probably leading to potential bias to our paper. Second, although randomized controlled trials were covered, the covered major studies had some intrinsic and methodological shortcomings: (1) only 10 trials reported information generated by randomization. (2) Some studies did not report the blinded process or had unclear results, which might lead to unintentional or intentional biases to their results and reduce the credibility of their research conclusions. Three blinds are required in further trials. Third, AS is a chronic disease that requires lifelong treatment. The long-term safety and efficacy of drugs are the key to determine the clinical efficacy of therapeutic drugs. However, the treatment period of our paper was between 8 and 24 weeks. The long-term safety of TG was not found for AS because the duration of treatment of the covered studies was short, and no dropouts were revealed in a considerable number of the covered studies. Fourth, the dosage, administration methods, and course of TG treatments differed remarkably in the major randomized controlled trials. This clinical heterogeneousness might jeopardize the feasibility of the results of this paper. Fifth, we searched researches published in Chinese or English repositories only, so the potentially relevant randomized controlled trials published in other languages might be excluded. Besides, all randomized controlled trials covered here were made in China, a potential limitation to the generalizability of the results of this paper. Sixth, our paper had low quality of evidence due to the high risk of bias and inconsistency. Therefore, it is necessary to further carry out multicenter randomized controlled trials of high-quality TG in the treatment of AS worldwide, so the data can provide a worldwide reference.

### 4.5. Implications for Research

Important ideas that may advance research in this area are revealed here. First, strategies to improve the methodological quality of randomized controlled trials are urgently needed. In the future, we recommend guidelines, covering CONSORT 2010 statement [43], that are needed to establish and report on randomized controlled trials of TG. Second, although TG treatment was found to be safe for AS patients in the analyzed studies, the safety of TG on AS remains to be further studied. The standard reporting format of ADR was developed [44], and it was suggested to pay close attention to ADR reports of TG. Third, to finally understand the long-term safety of triglycerides in patients suffering from AS, clinical trials and studies with long follow-up periods are recommended. As indicated by the results of this paper, TG can be used as an alternative treatment for patients suffering from AS, but further large-scale clinical studies are needed. In addition, the long-term safety, efficacy, and optimal dose of TG to treat AS should be explored.

### 4.6. Possible Mechanisms

Ji et al. [17] made a case-control investigation, reporting the ability of TG in improving the symptoms and signs of patients suffering from AS effectively on the basis of serum biomarker examination. The mechanism may be related to anti-inflammatory effects, inhibition of novel bone from being formed, and possible bone protective impact. Another study reported by them [45] showed that TG was efficient for the treatment of AS patients, and its mechanism of action may be correlated with the upregulation of cluster of differentiation (CD)4^+^CD25^+^ CD127^low^ regulatory T cells and the downregulation of interleukin- (IL-) 17 levels in the peripheral blood. Furthermore, Zhang et al. [46] employed network pharmacology for the analysis of the active ingredients, the prediction of core TG targets, and pathways to treat AS, then preliminarily demonstrated the mentioned targets with the use of molecular docking. The enrichment investigation indicated that TG participates in various biological processes, covering acute inflammatory response, cell-matrix adhesion regulation, cell-cell adhesion, on the basis of TNF-*α*, nuclear factor-kappa B (NF-*κ*B), and other signal pathways. However, the specific mechanism should be further clarified further *in vivo* and *in vitro* tests are required.

## 5. Conclusion

In brief, we showed supporting evidence that at least to an extent TG as adjuvant therapy or monotherapy for AS reduces the BASDAI, BASFI, SP-VAS, serum CRP, and ESR without increasing the ADR. Furthermore, TG treatment visibly improved the overall effective rate in AS. Therefore, the findings of this paper proved TG as a possible candidate in terms of AS treatment. Nevertheless, given the heterogeneity along with small sample size, more randomized controlled trials with large multicenter and high quality should be performed to deepen the benefits of TG to treat AS.

## Figures and Tables

**Table 1:** The characteristics of the included studies.

Study	Study design	Sample size	Sample and characteristics (male/female; mean age, year)		Interventions		Course of treatment	Outcome index
			EG	CG	EG	CG		
Wang 2018	RCT	80	40 (31/9); 27.13 y	40 (33/7); 26.84 y	TG (20 mg, tid)	LEF (first week: 10 mg, tid; from second week to last: 10 mg, bid)	NA	ADR, ER
Liang 2017	RCT	48	24 (14/10); 43.21 y	24 (14/10); 42.15 y	TG (20 mg, tid)	SSZ (first week: 250 mg, tid; second to third week: 500 mg, tid; from fourth week to last: 1000 mg, bid)	8 weeks	ER, SP-VAS
Song 2014	RCT	59	39 (31/8); 35.95 y	20 (14/6); 36.81 y	TG (20 mg, tid)	SSZ (first week: 250 mg, tid; second week: 500 mg, tid; from third week to last: 1000 mg, bid)	8 weeks	BASDAI, ADR
Jia 2014	RCT	58	29 (26/3); 25.7 y	29 (27/2); 25.8 y	TG (20 mg, tid)	LEF (first week: 10 mg, tid; from second week to last: 10 mg, bid)	24 weeks	BASDAI, SP-VAS, ESR, ADR
Liu 2014	RCT	50	25 (14/11); 45.6 y	25 (13/12); 43.8 y	TG (20 mg, tid)	SSZ (first week: 250 mg, tid; second to third week: 500 mg, tid; from fourth week to last: 1000 mg, bid)	8 weeks	ADR, ER
Luo 2013	RCT	121	60 (40/10); 35.1 y	61 (52/9); 33.6 y	TG (20 mg, tid)	LEF (weight > 50 kg: first three days 50 mg/d, then 50 mg/w; weight < 50 kg: first three days 40 mg/d, then 40 mg/w)	8 weeks	ADR, ER, BASDAI, BASFI, SP-VAS, ESR, CRP
Ji 2011	RCT	65	45 (NA); NA	20 (NA); NA	TG (20 mg, tid)	SSZ (first week: 250 mg, tid; second week: 500 mg, tid; from third week to last: 1000 mg, bid)	8 weeks	ADR, ER, BASDAI, BASFI, SP-VAS, ESR, CRP
Dong 2021	RCT	92	46 (28/18); 40.38 y	46 (25/21); 39.67 y	TG (1 mg/kg, tid) + CG	SSZ (first week: 250 mg, tid; second week: 500 mg, tid; from third week to last: 1000 mg, bid)	24 weeks	ER, BASDAI, BASFI
Liu 2020	RCT	60	30 (21/9); 38.09 y	30 (22/8); 37.42 y	TG (1 mg/kg, tid) + CG	ETA (25 mg subcutaneous injection, biw)	12 weeks	ER, BASDAI, BASFI, ESR, CRP
Shen 2020	RCT	94	47 (27/20); 34.02 y	47 (30/17); 32.11 y	TG (1 mg/kg, tid) + CG	ETA (25 mg subcutaneous injection, biw)	24 weeks	ER, ADR
Gu 2020	RCT	80	40 (29/11); 37.79 y	40 (28/12); 37.74 y	TG (0.5 mg/kg, tid) + CG	SSZ (first week: 1000 mg, tid; second week to last: 6000 mg, tid)	8 weeks	BASFI, ESR, CRP
Luo 2019	RCT	86	43 (26/17); 36.28 y	43 (28/15); 35.91 y	TG (20 mg, tid) + CG	ETA (25 mg subcutaneous injection, biw)	12 weeks	ADR, ER
Ji 2019	RCT	92	46 (26/20); 35.58 y	46 (24/22); 34.81 y	TG (1 − 1.5 mg/kg, tid) + CG	SSZ (first week: 250 mg, tid; second week: 500 mg, tid; from third week to last: 1000 mg, bid)	24 weeks	ER, BASDAI, BASFI, ESR, CRP, ADR
Xiong 2017	RCT	106	53 (27/26); 31.3 y	53 (28/25); 31.6 y	TG (1 − 1.5 mg/kg, tid) + CG	SSZ (first week: 250 mg, tid; second week: 500 mg, tid; from third week to last: 1000 mg, bid)	NA	ER, ESR, CRP
Zhou 2016	RCT	95	50 (29/21); 36.96 y	45 (25/20); 37.46 y	TG (20 mg, tid) + CG	ETA (25 mg subcutaneous injection, biw)	8 weeks	SP-VAS, BASDAI, BASFI, ESR, CRP, ADR

RCT: randomized controlled trial; EG: experimental group; CG: control group; TG: Tripterygium glycosides; LEF: leflunomide; SSZ: sulfasalazine; ETA: etanercept; BASDAI: Bath Ankylosing Spondylitis Disease Activity Index; BASFI: Bath Ankylosing Spondylitis Functional Index; CRP: C-reactive protein; ESR: erythrocyte sedimentation rate; SP-VAS: Spinal Pain Visual Analog Score; ADR: adverse drug reaction; ER: effective rate; NA: not available; bid: twice a day; tid: three times a day; biw: twice a week; y: year.

**Table 2:** Subgroup analysis.

Outcome	Subgroup factor	Number of study	Cases (EG/CG)	*I* ^2^ (%)	Heterogeneity (*P*)	Pooling model	*Z* test (*P*)
BASDAI		8	336/296	87.7	<0.0001	Random	<0.0001
	Course of treatment						
	≤8 weeks	4	185/145	55.3	0.082	Random	<0.0001
	>8 weeks	4	151/151	75.6	0.006	Random	<0.0001
	Sample size						
	≤60	3	98/79	92.6	<0.0001	Random	0.006
	>60	5	238/217	80.4	<0.0001	Random	<0.0001
	Age						
	≤35	3	134/110	94.0	<0.0001	Random	0.042
	>35	5	202/186	73.5	0.005	Random	<0.0001
	Control medication						
	SSZ	4	176/132	75.8	0.006	Random	<0.0001
	LEF	2	89/90	96.9	<0.0001	Random	0.164
	ETA	2	80/75	84.3	0.012	Random	0.001
	TG dosage						
	20 mg, tid	5	223/175	92.0	<0.0001	Random	0.002
	1 mg/kg, tid	3	113/121	0	0.525	Fixed	<0.0001
SP-VAS		5	224/195	88.0	<0.0001	Random	0.002
	Course of treatment						
	≤8 weeks	4	179/150	80.2	0.002	Random	<0.0001
	>8 weeks	1	45/45	—	—	Fixed	0.824
	Sample size						
	≤60	2	53/53	96.5	<0.0001	Random	0.347
	>60	3	171/142	45.5	0.160	Fixed	<0.0001
	Age						
	≤35	3	150/126	81.2	0.005	Random	0.086
	>35	2	74/69	82.5	0.017	Random	0.001
	Control medication						
	SSZ	2	69/44	92.7	<0.0001	Random	0.082
	LEF	2	89/90	90.6	0.001	Random	0.358
	ETA	1	50/45	—	—	Fixed	<0.0001

EG: experimental group; CG: control group; TG: Tripterygium glycosides; LEF: leflunomide; SSZ: sulfasalazine; ETA: etanercept; BASDAI: Bath Ankylosing Spondylitis Disease Activity Index; SP-VAS: Spinal Pain Visual Analog Score.

**Table 3:** GRADE evidence profile.

Quality assessment							No. of patients		Effect		Quality	Importance
No. of studies	Design	Risk of bias	Inconsistency	Indirectness	Imprecision	Other considerations	Tripterygium glycosides	Control	Relative (95% CI)	Absolute		
BASDAI (better indicated by lower values)												
8	Randomized trials	Serious^1^	Serious^2^	No serious indirectness	No serious imprecision	None	345	297	—	SMD 0.95 lower (1.48 to 0.42 lower)	⊕ ⊕ *ΟΟ* low	Critical
BASFI (better indicated by lower values)												
7	Randomized trials	Very serious^1,3^	No serious inconsistency	No serious indirectness	No serious imprecision	None	317	288	—	SMD 1.66 lower (1.85 to 1.48 lower)	⊕ ⊕ *ΟΟ* low	Critical
CRP (better indicated by lower values)												
7	Randomized trials	Very serious^1,4^	No serious inconsistency	No serious indirectness	No serious imprecision	None	324	295	—	SMD 0.76 lower (0.93 to 0.6 lower)	⊕ ⊕ *ΟΟ* low	Critical
ESR (better indicated by lower values)												
8	Randomized trials	Serious^1^	No serious inconsistency	No serious indirectness	No serious imprecision	None	353	324	—	SMD 0.91 lower (1.07 to 0.75 lower)	⊕ ⊕ ⊕*Ο* moderate	Critical
SP-VAS (better indicated by lower values)												
5	Randomized trials	Very serious^1,4^	Serious^2^	No serious indirectness	No serious imprecision	None	208	179	—	SMD 0.98 lower (1.7 to 0.26 lower)	⊕*ΟΟΟ* very low	Important
ER												
11	Randomized trials	Very serious^1,3^	No serious inconsistency	No serious indirectness	No serious imprecision	None	380/459 (82.8%)	284/435 (65.3%)	RR 1.26 (1.17 to 1.36)	170 more per 1000 (from 111 more to 235 more)	⊕ ⊕ *ΟΟ* low	Critical
								69.8%		181 more per 1000 (from 119 more to 251 more)		
ADR												
9	Randomized trials	Very serious^1,3^	No serious inconsistency	No serious indirectness	No serious imprecision	None	61/364 (16.8%)	45/315 (14.3%)	RR 1.22 (0.85 to 1.74)	31 more per 1000 (from 21 fewer to 106 more)	⊕ ⊕ *ΟΟ* low	Critical
								10%		22 more per 1000 (from 15 fewer to 74 more)		

GRADE working group grades of evidence: high quality: further research is very unlikely to change our confidence in the estimate of effect. Moderate quality: further research is likely to have an important impact on our confidence in the estimate of effect and may change the estimate. Low quality: further research is very likely to have an important impact on our confidence in the estimate of effect and is likely to change the estimate. Very low quality: we are very uncertain about the estimate. ^1^Some of the included studies did not report the implementation of blinding. ^2^Heterogeneity (*I*^2^ > 50%, *P* < 0.05) was found. ^3^Some of the included studies did not report the details of random protocol. ^4^Some of the included studies lack of allocation concealment.

## Data Availability

Previously reported data were used to support this study. These prior studies and datasets are cited at relevant places within the text as references [24–38].

## Abbreviations

AS: Ankylosing spondylitis
TG: Tripterygium glycosides
RCT: Randomized controlled trial
ESR: Erythrocyte sedimentation rate
CRP: C-reactive protein
SP-VAS: Spinal Pain Visual Analog Score
BASFI: Bath Ankylosing Spondylitis Functional Index
BASDAI: Bath Ankylosing Spondylitis Disease Activity Index
ADR: Adverse drug reaction
HLA: Human leukocyte antigen
TNF: Tumor necrosis factor
DMARDs: Disease-modifying antirheumatic drugs
NSAIDs: Nonsteroidal anti-inflammatory drugs
SSZ: Sulfasalazine
LEF: Leflunomide
PRISMA: Preferred Reporting Items in terms of Systematic Review and Meta-Analysis
AMSTAR: Assessment of systematic review methodological quality
RR: Relative risk
SMD: Standard mean difference
ER: Effective rate
RoB: Risk of bias
CD: Cluster of differentiation
IL: Interleukin.

## Supplementary Materials

Supplementary Materials PRISMA_2020_checklist and search strategy were provided as supplementary material. (Supplementary Materials).
