# Supplementary material for: Protective Effect and Possible Mechanisms of Tripterygium Glycosides in Patients with Ankylosing Spondylitis: A Systematic Review and Meta-Analysis
Source: Oxid Med Cell Longev. 2022 Mar 3;2022:9374895. doi: 10.1155/2022/9374895 (PMC8913062; doi:10.1155/2022/9374895)
Supplement: Supplementary Materials — PRISMA_2020_checklist and search strategy were provided as supplementary material. (Supplementary Materials). [file 9374895.f1.zip › Search strategy.docx]

The strategy for searching within the English database covered: ([“Tripterygium wilfordii Hook F”] OR [“Tripterygium wilfordii”] OR [“Tripterygium glycosides”] OR [“Tripterygium”] OR [“thunder god vine”]) AND ([“Ankylosing spondylitis”] OR [“AS”]) AND ([“random control trials”] OR [“randomized controlled trial”]). For the Chinese databases, we employed free text terms, covering ([“lei gong teng (i.e., Tripterygium wilfordii Hook F in Chinese)”] OR [“lei gong teng duo gan (i.e., Tripterygium glycosides in Chinese)”]) AND “qiang zhi xing ji zhu yan (i.e., ankylosing spondylitis in Chinese)” AND “sui ji dui zhao shi yan (i.e., randomized controlled trial in Chinese)”.
